# Proposal for future diagnosis and management of vascular tumors by using automatic software for image processing and statistic prediction

**Published:** 2015

**Authors:** MD Popescu, L Draghici, I Secheli, M Secheli, M Codrescu, I Draghici

**Affiliations:** * ”Carol Davila” University of Medicine and Pharmacy, Bucharest, Romania; ** Department of Pediatric Surgery, “Maria Sklodowska Curie” Clinical Emergency Hospital for Children, Bucharest, Romania; *** Department of General Surgery, “Sf. Ioan” Clinical Emergency Hospital, Bucharest, Romania

**Keywords:** infantile hemangioma, computer-aided diagnosis, esthetic sequels, color-coded duplex sonography

## Abstract

**Rationale.** Infantile Hemangiomas (IH) are the most frequent tumors of vascular origin, and the differential diagnosis from vascular malformations is difficult to establish. Specific types of IH due to the location, dimensions and fast evolution, can determine important functional and esthetic sequels. To avoid these unfortunate consequences it is necessary to establish the exact appropriate moment to begin the treatment and decide which the most adequate therapeutic procedure is.

**Objective.** Based on clinical data collected by a serial clinical observations correlated with imaging data, and processed by a computer-aided diagnosis system (CAD), the study intended to develop a treatment algorithm to accurately predict the best final results, from the esthetical and functional point of view, for a certain type of lesion.

**Methods and Results.** The preliminary database was composed of 75 patients divided into 4 groups according to the treatment management they received: medical therapy, sclerotherapy, surgical excision and no treatment. The serial clinical observation was performed each month and all the data was processed by using CAD.

**Discussions.** The project goal was to create a software that incorporated advanced methods to accurately measure the specific IH lesions, integrated medical information, statistical methods and computational methods to correlate this information with that obtained from the processing of images. Based on these correlations, a prediction mechanism of the evolution of hemangioma, which helped determine the best method of therapeutic intervention to minimize further complications, was established.

**Abbreviations:** Infantile Hemangiomas = IH, Computer Aided Diagnosis = CAD, Society for the Study of Vascular Anomalies = ISSVA, Color-coded duplex sonography = CCDS

## Introduction

The spectrum of vascular anomalies includes two distinct entities: malformations and vascular tumors. Vascular tumors are represented by infantile hemangiomas (IH), the most frequent type of benign tumor in children and other types of hemangiomas (hemangioendothelioma). IH are benign vascular tumors characterized by the proliferation of endothelial cells and which, during the stages of development, present an increase of the number of sanguine vessels starting from the periphery towards the center of the formation. Because of this vascular development, the dimension and the texture of the tumor are constantly changing, making it difficult to diagnose and to elect the therapeutic methods [**1**].

The majority of vascular tumors appear in the first weeks of life; only 30% are present at the moment of birth. The incidence in newborn is of 1, 1 – 2, 6% and significantly increases up to 12% in the first year of life [**2**]. IH are more frequently met in feminine sex and have an increased incidence with premature infants (30% frequency). In terms of localization, 56% are at the head and neck level, 23% at the body level, 19% affecting extremities and 2% in the genital area [**3**].

Until present, the therapeutical options have been multiple and have varied significantly in connection with the equipment of the treatment center and the experience of the physician. Due to the fact that the majority of IH do not present complications during the course of evolution and involute spontaneously, a treatment by intervention is not usually indicated. One can relate to therapeutical methods in case of hemangiomas that present complications (ulcerations, bleedings), for “dangerous hemangiomas”, or for those with rapid proliferation. The therapeutical principles currently applicable are the following: 1. Stopping the proliferation phase; 2. Speeding the regression of specific important hemangiomas; 3. Preventing, but also treating the functional problems; 4. The sooner the proliferation phase is ended, the better the final result after regression.

The following treatment methods are employed at present: laser therapy, surgical excision, medical therapy, sclerotherapy, waiting and surveillance. Only four of these treatments can be performed in our clinic, and we have hopes of implementing the laser therapy in the next year, as well [**2**]. 

## Materials and methods

**Fig. 1:**
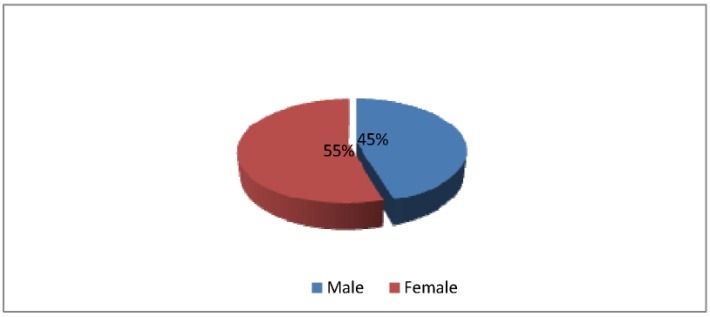
Sex distribution of IH

**Fig. 2:**
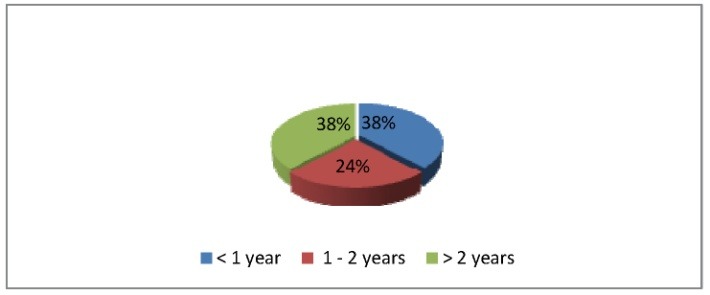
Age distribution of patients with IH at the beginning of treatment

75 cases of IH who received one of the four treatment methods presented before were registered in 2013, in the Department of Pediatric and Orthopedic Surgery of “M.S. Curie Hospital”, Bucharest. 39 cases were new patients, with the average age of the first clinical exam at 4 months, and 36 cases were follow-ups of previously known cases. 54,6% of the patients were females, 45,33% males (**Fig. 1**) and 38% of them had the age under one year, 24% between one and two years and 38% over 2 years (**Fig. 2**).

**Fig. 3:**
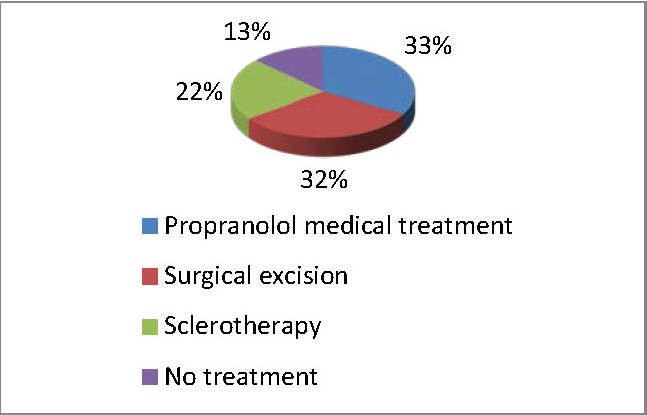
Percentage of patients treated by using one of the four types of therapy

Of these patients, 17 cases were treated by sclerotherapy, using Bleomycine, 24 cases underwent surgery for excision of the IH, 25 received medical therapy with Propranolol and 10 cases did not receive any treatment (**Fig. 3**). The last treatment was not yet available in our clinic, thus all the data collected refer only to four therapeutic measures.

**Fig. 4:**
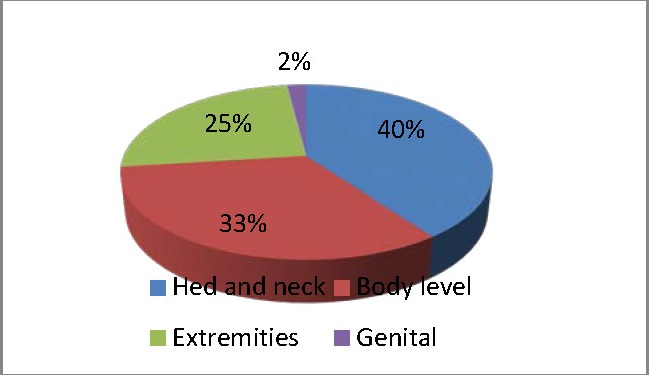
Localization of IH in our study group

The localization of the IH in our study group was slightly different from the one found in literature, having fewer cases affecting the head and neck area (40%) and more involving the body level (33%) and extremities (25%) (**Fig. 4**).

We were in the process of creating a database of photographic, ultrasound images, video recording, clinical and anamnestic data obtained as a result of a careful examination and surveillance. The acquisition of such items continued during the entire project, each patient having at least 5 different examination sheets. The follow up period was of 1 month or 3 months after a procedure and all the measurements and images were collected by using the same devices.

Among the technical challenges that occurred, the most important was represented by the poor quality of the digital images or of the video recordings. This problem occurred especially because of the patients. It should be mentioned once again that the study had as subject infants and small children, who had a diminished capacity of complying with the logistic requirements for the recordings. The agitation of the patient during the clinical examination and the imagistic investigation led to unclear photos, inadequate video recordings and inexact ultrasound recordings. As a measure of diminishing this impediment, a close co-operation with the family of the patients was required, together with a permanent exchange of information. The challenge was solved by centralizing a sufficient number of medical cases and associated images. The number of images collected for each studied period was sufficiently high in order to choose the most relevant data that were sent for processing.

The clinical documentation was the input data set that was taken by the engineering and mathematical researchers, which worked together on two successive levels: on one hand, the information extracted from the images was be processed, on the other hand, the results of this processing were correlated (i.e. put into a mathematical correlation) with the clinical observations. The result of this correlation allowed the obtaining of a prediction method that was made available to the medical side, so that with new information from imaging (and of course by using the developed software) the most likely evolution was anticipated. The clinical results subsequently obtained showed the attained level of accuracy of the developed prediction method and, either were a confirmation of it, or generated an additional step in refining the method.

## Discussions

At present, the pathology benefits of very little study in our country and there is no well-defined protocol to supervise in time or to treat this affection.

Until the beginning of the ‘80s, the classification described both the vascular tumors and the malformations as “vascular birth signs” [**4**]. In 1982, the first classification of the vascular anomalies was established by separating them into hemangiomas (vascular tumors) and malformations. The key factor of this separation was the identification of an accentuated proliferation phase of the vascular tumors followed by an involution phase. In 2012, a new classification was proposed, taking into consideration the clinical aspect and the degree of tissue damage: localized hemangiomas, segmented hemangiomas, undetermined and multifocal hemangiomas [**5**].

The project proposal was based upon the development of advance methods for the precise and objective measuring of specific lesions of infantile hemangiomas and upon the prediction of the evolution of the hemangiomas in order to determine the best therapeutical solution. The natural evolution of IH was unique and presented 5 stages of development, as described in **Table 1**. The clinical characteristics of each stage were co-related with the data registered with the help of a Doppler ultrasound (CCDS) which codified the colors [**2**].

**Table 1:** The 5 stages evolution of IH [**2**]

Stage	Clinic	CCDS
1.Prodromal	Red/ white spot, telangiectasia	Structureless;
2.Initial	Increasing thickness and induration	Hipervascularization beginning at edges
3.Proliferation	Bright red cutaneous, indurated skin, growth of thickness, infiltrated margins, rapid increase in size	Increasing intratumoral hyperperfusion; center vessel density; one notices vessels feeding the tumor
4.Maturation	Color becomes light red or purple; possible central exulceration; decreasing growth	Declining central vessel density; increasing ectatic drainage veins; declining arterialization of drainage veins; central increasing hypersonore
5.Regression	Hypopigmentation; wrinkled skin the induration area remains subcutaneous, palpable; thickness observed in peripheral venous drainage	Loss of specific structure; the disappearance of almost all central vessels with residual tumor feeding vessels; residual drainage vessels in the periphery

Besides the growth speed of the IH, their localization was the most important criteria that had to be followed as a therapeutical attitude. The therapy generated systemic or local adverse reactions, especially in the presence of disfigurative scars. Because of this reason, the interventional treatment was limited in case of IH that determined important functional or esthetical complications. Out of the group of problematic IH which necessitated treatment, the following could be identified: the hemangiomas of the facial area (periorbital, perioral, ears area, lips area or nose area), the anal and genital area (vulva, urethra, anus), and the ones which had an accelerated, diffuse and infiltrative proliferation, independently of the affected region [**2**].

**Fig. 5:**
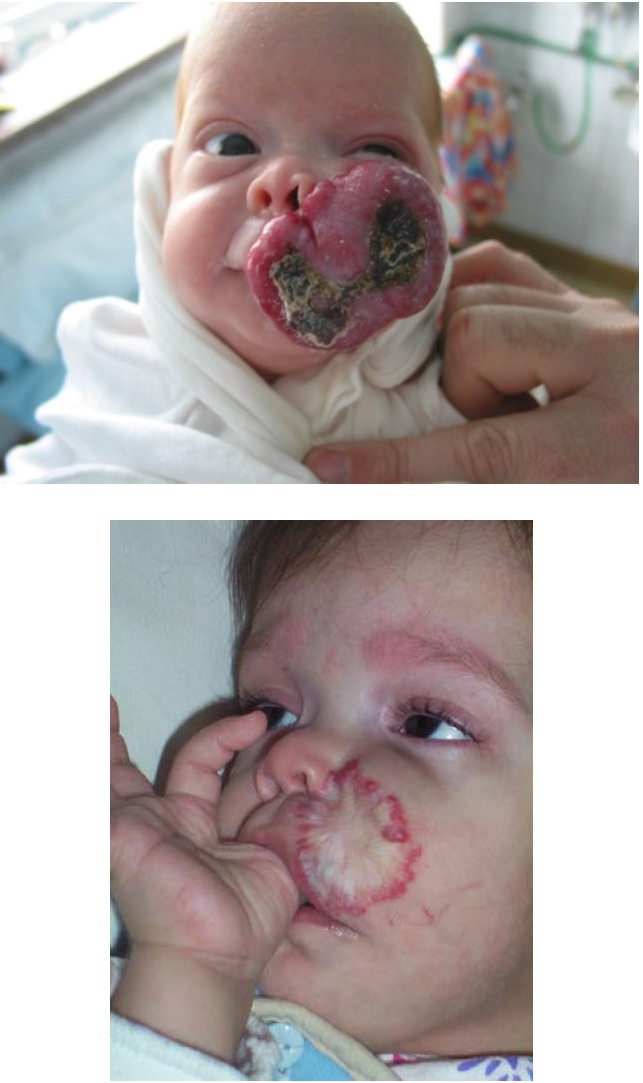
a) IH age 1 month b) Same patient age 2 years

Due to the possibility of the IH of spontaneously regressing, waiting and watching remained viable therepeutical methods, in the case of lesions small and without complications localized in non-problematic areas of the body. IH of the head and neck area were the most frequent and were also the most disfigurative because of the affected areas (**Fig. 5 a,b**), as well as the hemangiomas with an important dimension, both in length and in depth. These made the taking of a rapid decision regarding the best therapeutical method, imperative.

In the usual clinical practice, the evolution of an IH was estimated only by the simple measuring with a ruler of the dimensions of the lesion during the periodical clinical examinations. The possible commencement of the regression stage, as well as the regression degree were estimated only by the visual inspection of the affected area and the registration of the surface percentage which became more blurred, in comparison with the rest of the IH which was still reddish. Of course, this way of following the evolution is totally imprecise, as measuring errors may occur, and the estimation of the regression percentage is very subjective [**6**].

At present, there are no modalities that can enable the prediction of the way in which an IH will evolve in time, from the clinical point of view and by taking into consideration the complications that might occur. Moreover, there are no well-known characteristics that may affect the speed of involution of the infantile hemangiomas and which may predict its ending [**2**], aspects that underline the imperative necessity of identifying a method of detection and prediction in this field [**1**].

Advances in computer science and artificial intelligence have a direct impact on the interpretation of medical images [**7**], computer aided diagnosis (CAD) is about to become part of clinical routine in more and more areas of medical practice [**8**]. Computer Aided Diagnosis aims to provide an answer from the computer as a second opinion to help the physician detect anomalies and quantify the disease progress. For the lesion characterization, the physician’s interpretation errors can occur due to complex anatomical structures, specific differences between evolution stages and finally its abilities. CAD systems are designed to facilitate the stages of detection and characterization of the lesion by enhancing the physician’s capabilities and reducing the time required for a precise diagnosis [**7**,**9**,**10**].

Based upon the clinical data collected by serial clinical observation, co-related to imagistic data, the project intended to develop an algorithm of treatment that accurately predicted the best final results in terms of esthetics and functionality for a specific type of lesion. The algorithm of treatment was transposed by an easy to access medical software and of real use for all medical personnel who diagnose this pathology. This automatic image processing software was able to accurately and objectively determine what stage of development the IH was (proliferation or regression) in, as well as to provide an exact percentage of the area of regression. To achieve this, one would use and/ or develop modern methods of medical image processing and intelligent algorithms for aid in diagnosis (CAD). The software integrated the medical information resulting from clinical observations and incorporated statistical methods and/ or computational methods inspired by nature (i.e. nonlinear dynamics, artificial cellular neural networks) to correlate this information with the results of image processing. Based on these correlations, one would establish a prediction mechanism for the evolution of hemangiomas, which will help choosing the best therapeutic intervention method. By using this medical software, family physicians, neonatologists, pediatrics or pediatric surgeons would be able to decide which the appropriate treatment is and what would most likely be the best attitude for the given type of IH, from the first contact with the patient. Thus, one proposed the implementation of a new method for the prevention of disfiguring complications, both aesthetically and functionally, by determining the exact type of interventional method to be applied at the right moment of time.

However, although computer-aided diagnosis has become a part of the clinical assessment in certain medical areas, such as detection of breast cancer through mammography, its applications in various other skin lesions obtained by different modalities is still at an early stage.

## Conclusions

The results of the study implied real health benefits for both patients and the health system, receiving a minimization of costs per patient in this condition. By decreasing the number of procedures and defining a single therapeutic strategy for the treatment of a patient, amounts could be saved and consequently allocated for the benefit of other needs. Moreover, if the aesthetic and functional result is maximized, the resources allocated for the patient’s reintegration into society will be saved, which is more costly if the degree of failure is higher. Decreasing the disease follow up period, speeding the diagnosis, diminishing the hospitalization time, a faster social integration of the patients, are only a few benefits that such a study brings, not only for the patients, but also for the national and European medical system.

**Acknowledgement**

This paper is supported by the Sectorial Operational Program Human Resources Development (SOP HRD) 2007-2013, financed from the European Social Fund and by the Romanian Government under the contract number: POSDRU/159/1.5/S/137390.

This paper is supported by the Funding Application for Joint Applied Research Projects PN-II-PT-PCCA-2013-4

**Disclosures**

None
